# Ferromagnetic alloy for high-efficiency photovoltaic conversion in solar cells: first-principles insights when doping SnO_2_ rutile with coupled Eu–Gd

**DOI:** 10.1039/d1ra00088h

**Published:** 2021-02-10

**Authors:** A. Fakhim Lamrani

**Affiliations:** Nanomaterial and Nanotechnology Unit, E. N. S. Rabat, Energy Research Center, Faculty of Sciences, Mohammed V University in Rabat B. P. 1014 Morocco fakhim@um5.ac.ma

## Abstract

From results of first-principles all-electron full-potential augmented spherical-wave calculations within a generalized gradient approximation, a materials design for half-metallic ferromagnetic semiconductors based on (Eu,Gd)-doped SnO_2_ rutile is proposed. Moreover, their half-metallic ferromagnetic properties are homogenous and energetically stable for different crystallographic directions. Therefore, the interatomic exchange interaction between the spins of double impurity ions is a long-range ferromagnetic interaction that is sharply weakened when the distance between Eu–Gd increases. The double impurities most likely substitute adjacent Sn sites and result in strong ferromagnetic interactions by p–f hybridization between rare earth 4f and Op states. There is great interest in the configuration that has the lowest energy difference, where the double impurity substitutes the nearest neighbor Sn sites along the *z*-axis of SnO_2_ rutile. Generalized gradient approximation GGA and GGA+U calculations were performed. According to our revPBE-GGA calculations, the ferromagnetic compound is capable of absorbing 96% from the visible light. Furthermore, the transport properties at room temperature ensure excellent electrical conductivity, low thermal conductivity, and the most optimal figure of merit (*ZT*), which leads to high thermoelectric performance. As the latter are closely related to free flow charge carriers, we can subsequently predict that the ferromagnetic alloy will be able to be a great power source for highly effective photovoltaic conversion in solar cells. Further experimentation will be necessary to obtain confirmation of our *ab initio* predictions.

## Abstract 2

A material design of half-metallic ferromagnetic semiconductors based on (Eu, Gd)-doped SnO_2_ rutile is proposed for High-Efficiency Photovoltaic Conversion in solar cells.

## Graphical abstract

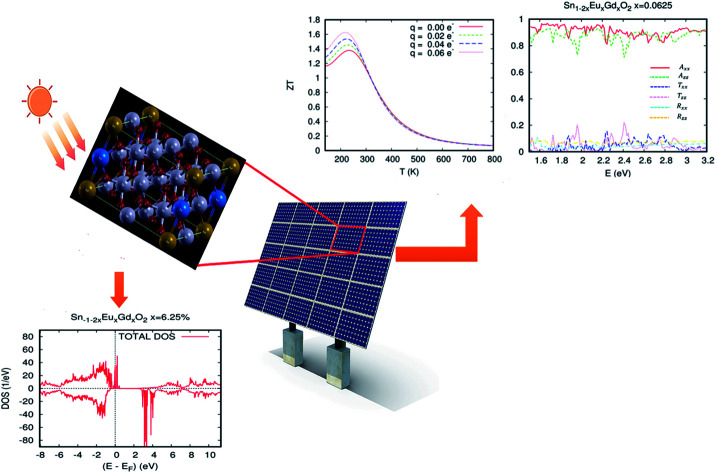

## 1 Introduction

Because of their many favorable attributes, oxide-based diluted magnetic semiconductors (DMS), particularly SnO_2_, are highly appropriate for constructing spintronic devices compared to non-oxide-based DMS.^1^ Because of the physical properties of tin oxide (SnO_2_), which include excellent optical transparency, metal-like conductivity, and high chemical stability, it is a highly multifunctional material with widespread applicability. Many great developments have also occurred in condensed matter physics for the creation of innovative functions.^2,3^

Since the observation of high-temperature ferromagnetism in Co-doped SnO_2_ films by Ogale *et al.*,^4^ and the development of a transparent ferromagnet with a Curie temperature of 610 K in Fe-doped SnO_2_,^5^ a large number of experimental and theoretical investigations have been performed using tin oxide doped with alkaline earth metals, transition metals, and rare earth ions.^6–19^ Although several research groups have delved into the study of spintronics coupled with optoelectronics, the field has been largely left untouched. Specifically, the production of solar cells based on ferromagnetic DMS has not yet been accomplished, and it may be a key material used in the future technology of solar cells. Indeed, ferromagnetic materials may display enhanced lifetimes of excited states due to spin-dependent transition selection rules, which have not yet been classified. For a photovoltaic energy conversion system, long lifetimes of excited states are important because they increase performance and provide the opportunity for photogenerated carriers to be collected. Currently, the main problem with photovoltaic solar cells is the conversion efficiency limitation,^20–24^ which remains due to the discord between the solar incident spectrum and the spectral absorption of the cell's material.

Some methods have been improved that consist of modifying the solar spectrum by a wavelength conversion process to render highly effective photovoltaic conversion. There are promising up- and down-conversion approaches that are realized by doping host matrices with rare earth elements.^25,26^

Because of the electronic structure and susceptibility of magnetic and optical properties of doped SnO_2_ to experimental conditions, it is preferable to conduct investigations utilizing theoretical calculations. Hence, the current study intends to examine the oxide-based semiconductor, SnO_2_ rutile doped with double rare-earth impurities (two different ones), rather than the traditional single impurities, using first-principles insights. Thus, after identifying the exact substitution sites of the couple Eu/Gd in the host matrix of SnO_2_ rutile, we attempted to understand the electronic, magnetic, and optical properties of (Eu,Gd)-doped SnO_2_ rutile. Our objective behind this research is twofold:

A. To supply a more accurate and complementary study on the electronic structure, and to describe the magnetic ground state of Sn_14_EuGdO_32_ using first-principles calculations based on revPBE-GGA. Then, we introduce results from revPBE-GGA+U calculations.

B. To understand the rendering of this ferromagnetic DMS under natural light, which is predominantly in the visible region, and its ability to participate in photovoltaic conversion in a solar cell.

## 2 Theoretical method

The calculations are based on density functional theory and the generalized gradient approximation (GGA)^27^ with the local density approximation parametrized according to Vosko, Wilk and Nusair (VWN).^28^ They were performed using the scalar-relativistic implementation of the augmented spherical wave (ASW) method (see ref. 29–31 and references therein). With the ASW method, the wave function is expanded in atom-centered augmented spherical waves, which are Hankel functions and numerical solutions of Schrödinger's equation, respectively, outside and inside the so-called augmentation spheres. In order to optimize the basis set, additional augmented spherical waves are placed at carefully selected interstitial sites. The choice of these sites as well as the augmentation radii were automatically determined using the sphere geometry optimization algorithm.^32^

Self-consistency was achieved by a highly efficient algorithm for convergence acceleration^33^ until the variation of the atomic charges was smaller than 10^−8^ electrons and the variation of the total energy was smaller than 10^−8^ Ryd. The Brillouin zone integrations were performed using the linear tetrahedron method with up to 6 × 6 × 9 *k*-points corresponding to 324 *k* points within the irreducible wedge.^31,34^ In the present work, we used a new full-potential version of the ASW method, which was implemented only very recently.^35^ In this version, the electron density and related quantities are given by a spherical harmonics expansion inside the muffin-tin spheres. In the remaining interstitial region, a representation in terms of atom-centered Hankel functions is used.^36^ However, in contrast to previous related implementations, it is unnecessary to use a so-called multiple-*k* basis set, and this allows for a very high computational speed of the resulting scheme.

SnO_2_ possesses tetragonal symmetry in the rutile structure. The rutile structure is characterized by two lattice parameters, *a* = 4.7373 Å and *c* = 3.1864 Å.^37^ The unit cell contains two metal atoms (Sn) at positions (0, 0, 0) and (1/2, 1/2, 1/2) and four oxygen atoms (O) at positions ± (*u*, *u*, 0; 1/2 + *u*, 1/2 − *u*, 1/2) with *u* = 0.306. Each Sn atom is in the central site of an octahedron, which is formed by four rectangular basal O atoms (O_1_) and two vertex O atoms (O_2_). Using the integer multiple representations of the primitive lattice vectors *a*, *b*, and *c* of the conventional SnO_2_ cell, the geometry of an undoped 2 × 2 × 2 supercell containing 48 atoms (Sn_16_O_32_) was determined. In order to model a composition Sn_1−*x*_Eu_*x*_O_2_, Sn_1−*x*_Gd_*x*_O_2_, and Sn_1−2*x*_Eu_*x*_Gd_*x*_O_2_ for *x* = 0.0625, one and two different Sn atoms are substituted. Two possible couplings [ferromagnetic (FM) and antiferromagnetic (AFM)] have been considered, between the double impurities.

## 3 Results and discussion

### 3.1 Electronic properties

**Fig. 1:**
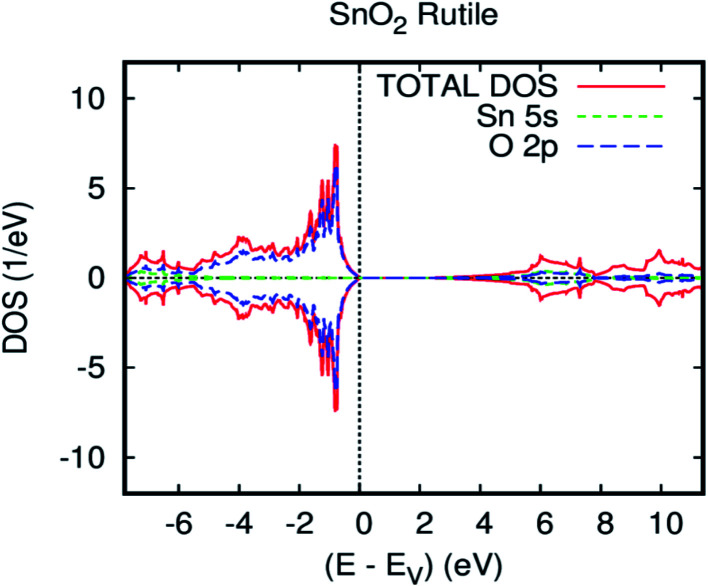
The GGA total and partial DOS of SnO_2_ rutile.

First, we confirmed the electronic structure of the parent material without any doping elements. Fig. 1 displays the revPBE-GGA total and partial density of states (DOS). The overall band structure of the present full-potential augmented spherical wave (FPASW) result is consistent with the existing results.^38,39^ It can be viewed that the majority-spin and minority-spin are symmetrical, which signifies that the tin oxide rutile is a nonmagnetic material. Indeed, the valence band is chiefly dominated by O-2p orbitals and is full, whereas the conduction band is principally formed by Sn-5s and is empty.

**Fig. 2:**
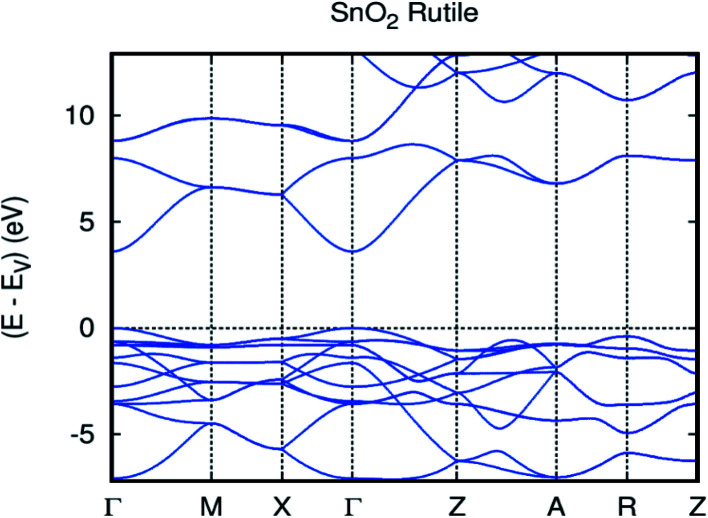
The GGA+U band structure of SnO_2_ rutile.

As usual in the GGA calculations, the obtained energy gap of 2.4 eV is underestimated. In fact, this shortcoming is well known, and compensation is applied by employing the GGA.^40^ In particular, we find a valence band width of 7.7 eV in accordance with the experimental data (7.5 eV mentioned in ref. 41) and the preceding first-principles technique (7.9 eV and 8.8 eV mentioned in ref. 42). The optimized bulk cell parameters for pure SnO_2_ with FPASW-GGA^43^ are in accordance with experimental values^37,44^ and other theoretical values.^45^ This clearly illustrates that our calculation is similar to the experimental ones. A strong correlation was applied to Op-electrons (U_O_ = 6.25 eV), and Fig. 2 pragmatically illustrates the GGA+U calculation of the band structure of SnO_2_ rutile. As a matter of fact, the GGA+U method satisfactorily^46^ enhances the energy gap in comparison with revPBE-GGA (3.6 vs*.* 2.4 eV), and provides stronger agreement with the experimental data.^47^ Additionally, the application of the Hubbard coefficient to the anion p states has a great impact on the correction of the gap energy.

### 3.2 Electronic and magnetic properties

**Fig. 3:**
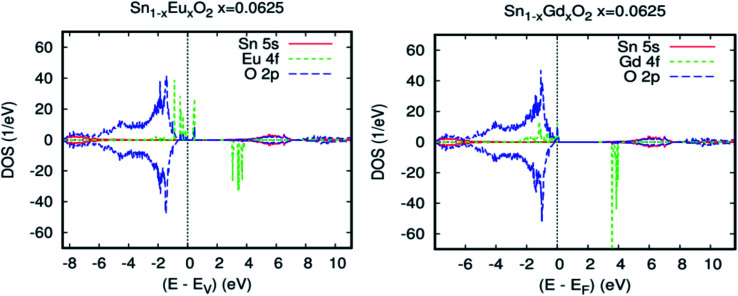
(a) Projected local DOS for Sn_0.9375_Eu_0.0625_O_2_, (b) Projected local DOS for Sn_0.9375_Gd_0.0625_O_2_. Fermi level is set at zero.

Prior to discussing the half-metallic ferromagnetic properties and high effectiveness of the photovoltaic conversion of SnO_2_ rutile doped with the couple Eu/Gd, we initially examined the electronic structure and the magnetic properties of SnO_2_ rutile doped with simple rare earth. Fig. 3(a) and (b) show the revPBE-GGA, the projected local DOS (PLDOS) for Sn_0.9375_Eu_0.0625_O_2_, and those for Sn_0.9375_Gdo_0.0625_O_2_. However, the PLDOS reveal that the europium impurity in the SnO_2_ matrix is a magnetic semiconductor. On the other side, we obtained a half-metallic magnet for Gd-doped SnO_2_ rutile. From Fig. 3(a), three groups of energy levels for Eu 4f in the spin-up states can be observed and partially filled. One exists at the valence band edge, the second is at the limit of the Fermi level, and the third one is above the Fermi level and is completely empty, while the spin-down-induced states, which are at the minimum of the conduction band, are completely empty. Therefore, the occupied electronic configuration of Eu-4f in Sn_0.9375_Eu_0.0625_O_2_ is f^5^, as presented in Table 1, and it is justifiable according to the analysis of the local magnetic moments of the impurities and the oxidation states from the charge carrier's occupancy in the 4f orbital. In contrast, it is clear from Fig. 3(b) that the Gd-based system is half-metallic [*i.e.*, for spin-up, states are available, whereas for spin-down, they are not available at the Fermi level]. A careful analysis of PLDOS indicates that the majority spin channel of Gd-4f is located at the top of the valence band and overlaps with O-2p orbitals at the Fermi level *E*_f_. Table 1 shows that the main part of the total magnetic moment comes from the gadolinium atom, which suggests that the Gd impurity doped in SnO_2_ has a magnetic configuration of 4f^6^.

**Table 1:** Total energy *E*_t_, energy gap *E*_g_, moment total *m*_t_ and partial *m*_O_, and *m*_RE 4f_ for SnO_2_ rutile doped with simple and double rare earth

Compounds	Sn_0.9375_Eu_0.0625_O_2_	Sn_0.9375_Gd_0.0625_O_2_	Sn_0.875_Eu_0.0625_Gd_0.0625_O_2_	Sn_0.875_Eu_0.0625_Gd_0.0625_O_2_	Sn_0.875_Eu_0.0625_Gd_0.0625_O_2_
	GGA	GGA	GGA	GGA+U_O_	GGA+U_O,Eu, and Gd_
*m*_4f_ (μ_B_)	5.2	6.14	*m*_Eu_ = 5.14; *m*_Gd_ = 6.31	*m*_Eu_ = 5.2; *m*_Gd_ = 6.34	*m*_Eu_ = 5; *m*_Gd_ = 6.8
*m*_O_ (μ_B_)	−0.034	−0.042	−0.11	−0.12	0.23
*m*_t_ (*μ*_B_)	5	6	11	11	13
*E*_g_ (eV)	0.33	2.54	2.65 (Γ to Γ)	3.85 (Γ to Γ)	1.65 (Γ to X)
*E*_t_ (Ryd)	−211836.148134	−212690.544629	222024.823722	222016.089220	222015.833623

To more precisely study the electronic structure and to describe the magnetic ground state of the couple europium–gadolinium-doped tin dioxide, we have: Sn_1−2*x*_Eu_*x*_Gd_*x*_O_2_ (*x* = 0.0625). In fact, we performed the revPBE-GGA calculation for modeling the effect of interatomic exchange interactions *via* following a simple model that consists of altering the distances between double rare earth ions in the supercell. Furthermore, three separations were realized, by the fixed Eu ion at the origin of the supercell, [000], and the other ion (Gd) moving along different crystal directions. The 3.2 Å corresponds to a double impurity substitution of the nearest neighbour Sn sites along the *z*-axis of SnO_2_ rutile, which indicates that Gd is placed at the site of [001], while 5.7 and 7.42 Å corresponds to farther Eu–Gd separations with Eu–O–Sn–O–Gd configurations along the plane [011], and diagonal direction [111], respectively (Table 2).

**Table 2:** The ferromagnetic and ferrimagnetic energy, the energy gap *E*_g_, and partial *m*_O_, *m*_RE 4f_*vs.* Eu–Gd distance in the unit cell for Sn_0.875_Euo_0.065_Gdo_0.065_O_2_

*d*_Eu–Gd_ (Å)	*d*_Eu–Gd_ = 3.2 Å	*d*_Eu–Gd_ = 5.7 Å	*d*_Eu–Gd_ = 7.42 Å
	Eu[000] → Gd[001]	Eu[000] → Gd[011]	Eu[000] → Gd[111]
*E*_Fero_ (Ryd)	−222024.828722	−222025.416400	−222024.056243
*E*_Feri_ (Ryd)	−222024.801777	−222025.407732	−222024.055023
*E*_g_ direct (eV)	2.65	2.764	2.81
*m*_Eu_ (*μ*_B_)	5.14	5.153	5.15
*m*_Gd_ (*μ*_B_)	6.31	6.221	6.20
*m*_0_ (*μ*_B_)	−0.11	−0.067	−0.053

**Fig. 4:**
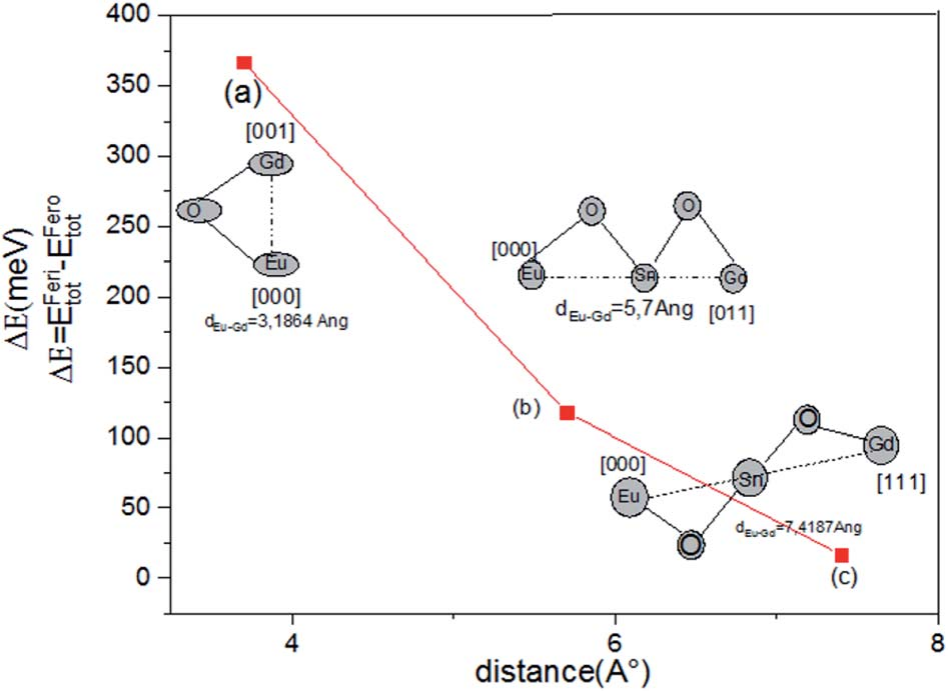
The energy difference between ferromagnetic and ferrimagnetic configurations *vs.* Eu–Gd distance in the unit cell for Sn_0.875_Eu_0.065_Gd_0.065_O_2_.

For each Eu–Gd separation, ferromagnetic (fero) and ferrimagnetic (feri) alignments of rare earth spins were considered. The total energy difference Δ*E*; (Δ*E* = *E*^feri^ − *E*^fero^) between these two alignments is a measure of interatomic exchange interaction. In Fig. 4, we plotted the energy difference between ferromagnetic and ferrimagnetic configurations *vs.* Eu–Gd distance in the unit cell for Sn_0.875_Euo_0.065_Gdo_0.065_O_2_. Ferromagnetic interaction between rare earth spins is favoured for all distance, and therefore, the exchange interaction between double impurity ions is a long-range ferromagnetic interaction, and it is weakened as the distance between Eu–Gd increases. Hence, the half-metallic ferromagnetic properties are homogenous in different crystallographic directions. As a matter of fact, the first nearest neighbour exchange interaction in the bonding direction Eu[000] → Gd[001] is the strongest, as shown in Fig. 4, where Δ*E* decreases sharply with *d*_Eu–Gd_. This suggests that the rare earth impurities might cluster together during sample growth, rather than distribute themselves evenly over the lattice.

**Fig. 5:**
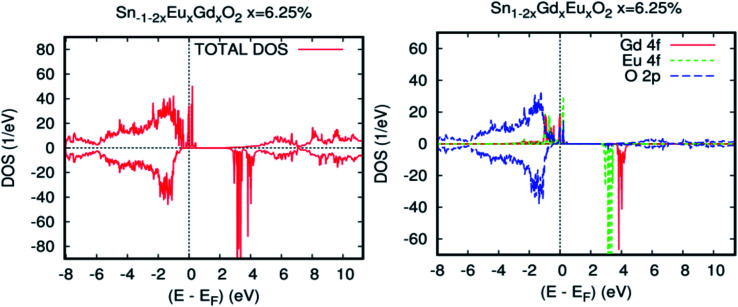
Calculated spin-resolved density of states (DOS) for Sn_0.9375_Eu_0.0625_Gd_0.0625_O_2_, for an Eu–Gd separation of 3.24 Å. (a) The total DOS, and (b) the partial DOS of neighbouring Sn-5s, O-2p, and RE-4f states. The Fermi level is set at zero.

For the farther Eu–Gd separation of 5.7 Å, where there is no O-2p atom between europium and gadolinium, this corresponds to the Eu–O–Sn–O–Gd configuration along the plane [011]. The coupling between spins of double rare earth impurity ions is still important in comparison with that mentioned in Co- and Fe-doped SnO_2_ rutile.^48^ In addition, for the [111] direction, the coupling sharply decreases because the Eu–Gd separation is farther and is not communicated by the O-2p diagonal direction. Fig. 5(a) and (b) show the total and partial density of states, respectively, calculated by revPBE-GGA, for an Eu–Gd separation of 3.2 Å. The occupied electronic configuration and PLDOS of the 4f orbital in the unit cell for Sn_0.875_Eu_0.065_Gd_0.065_O_2_ appear very similar to those of the single rare earth-doped cases illustrated in Fig. 3(a) and (b). This explains that the kinetic energy gain through the hopping of spin-polarized carriers between Eu and Gd ions does not appear to occur as long as there is no sign of charge transfer between Eu and Gd. Therefore, the double-exchange mechanism will not be effective in Sn_1−2*x*_Eu_*x*_Gd_*x*_O_2_.

On the basis of these data, we can explicate the robust ferromagnetism in the [001] and [011] directions by the p–f exchange mechanism, which weakens along the [011] plane. On the other side, the exchange interaction between spins of double rare earth in the Eu[000] → Gd[111] diagonal direction cannot be mediated by p–f hybridization because the value of Δ*E* is smaller in comparison with the other cases. Additionally, this study allows us to identify the exact substitution sites of the double rare earth impurities, Eu and Gd ions, in the SnO_2_ matrix. It was found that the lowest difference energy corresponded to the case where the couple Eu/Gd substituted the nearest neighbor Sn sites along the *z*-axis of the unit cell Sn_0.875_Eu_0.065_Gd_0.065_O_2_. Therefore, these two rare earth impurities are likely to be located at adjacent Sn sites, and these also interact through bridging O atoms, resulting in the p–f hybridization between the rare earth 4f and Op states.

Although this configuration has the lowest energy difference, in order to apply the Hubbard coefficient on the anion p states for reproducing the experimental band gap (3.60 eV) of the host system and treat the strongly correlated 4f electrons of double rare-earth impurities, the GGA+U approach was employed in addition to the generalized gradient approximation (GGA).^46^ The parameter used for the O-2p state is U_O_ = 6.25 eV. Because both gadolinium and europium have a rather similar 4f^7^, the values of U and *J* that reproduced the experimental observation of the splitting and which we used in our calculations are 7.62 and 0.68 eV, respectively.^49^ In addition, double-counting corrections were included within the fully localized limit (FLL).^46,50^

**Fig. 6:**
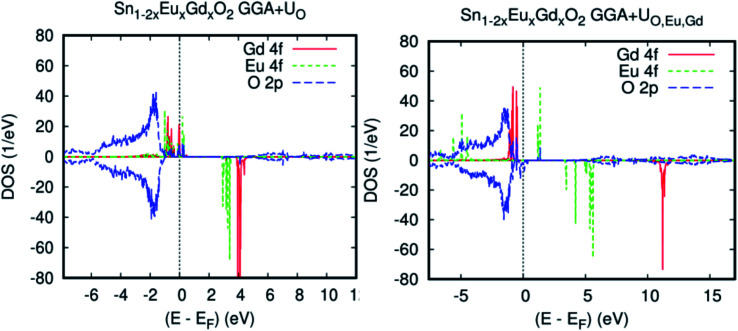
The GGA+U PLDOSs of Sn_0.9375_Eu_0.0625_Gd_0.0625_ O_2_ for an Eu–Gd separation of 3.24 Å. (a) The Hubbard coefficient was applied only to the anion p states of a host system. (b) The Hubbard coefficient was simultaneously applied to the anion p states and 4f electrons.

The spin density distribution for Sn_1−2*x*_Eu_*x*_Gd_*x*_O_2_, calculated by GGA+U (U is applied only on the anion p states of the host system), is very similar to that obtained by GGA, and therefore, there is an overall topological resemblance for both methods. The signature of a half-metallic solution is clear from the PLDOS presented in Fig. 6(a). The effect of the Hubbard parameter is clear in the energy gap, as shown in Table 1. Additionally, the 4f orbitals are slightly far from the top of the valence. Thus, both majority- and minority-spins of the coupled europium–gadolinium display a band gap, which indicates that the introduction of rare earth impurities does not destroy the semiconducting nature of the parent material.

In the case where we simultaneously applied the Hubbard coefficient to the anion p states and 4f electrons, surprisingly, (Eu,Gd)-doped SnO_2_ rutile displays a half-metallic characteristic behavior, but the spin-down from the anion O-2p states is polarized at Fermi level. Indeed, the semiconducting nature has been achieved by spin-up states of Eu-4f, as shown in Fig. 6(b). Hence, the 4f states are not available at the Fermi level, and the exchange splitting increases with the increase in the atomic number of rare earth elements. It was observed that the total magnetic moment of the system became large, with 13 Bohr magnetons, *μ*_B_, instead of 11 *μ*_B_, for previous cases (see Table 1).

### 3.3 Optical properties

**Fig. 7:**
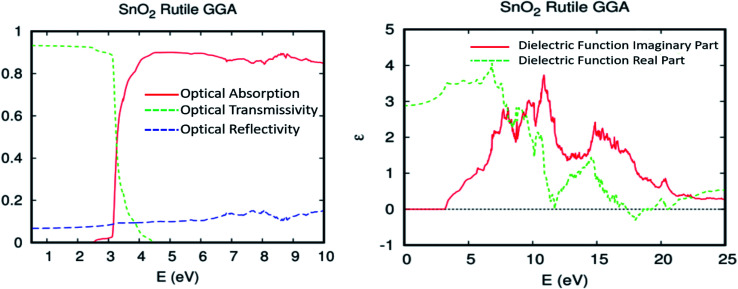
(a) The optical absorption, transmissivity, and reflectivity, and (b) the real and imaginary parts of the dielectric function for SnO_2_ rutile.

In this section, we examine the fundamental relationship between optical performance, electronic structure, and transport properties in order to better understand the highly effective photovoltaic conversion of SnO_2_ rutile doped with double rare earth impurities (two different ones). First, we conducted a study on the optical absorption, transmissivity, reflectivity, and dielectric function of undoped SnO_2_ rutile, as indicated in Fig. 7(a) and (b). The optical properties of the present FPASW on SnO_2_ rutile without doped elements altogether are in conformity with the experimental and theoretical data.^51–53^

Fig. 7(a) shows the optical transmissivity, absorption, and reflectivity of pure SnO_2_. It is clear that the value of transmissivity in the visible light that we have found is in the range of 89–93%, with the parent material exhibiting no response to visible light. The optical absorption coefficient is correctly defined by the rule 1 = *R* + *A* + *T*, where *R*, *A*, and *T* denote the optical reflectivity, optical absorption coefficient, and optical transmission coefficient, respectively. To analyze the optical properties in greater detail, we require a dielectric function: *ε*(*ω*) = *ε*_1_(*ω*) + i*ε*_2_(*ω*). The imaginary part *ε*_2_(*ω*) can be directly calculated from the full many-electron wave function. The real part *ε*_1_(*ω*) can be determined *via* a Kramers–Kronig transform. All the other optical constants will be deduced from *ε*_2_(*ω*) and *ε*_1_(*ω*), such as reflectivity *R*(*ω*) and absorption coefficient *α*(*ω*).^54–56^1
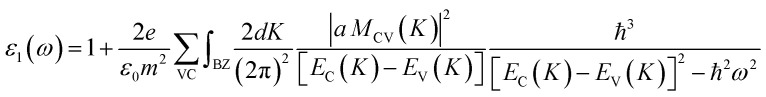
2
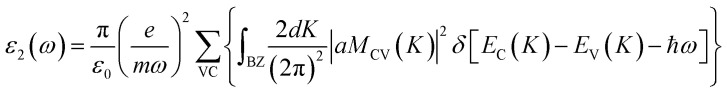
3
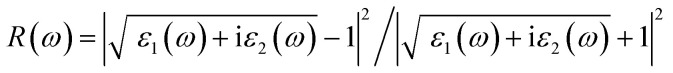
4
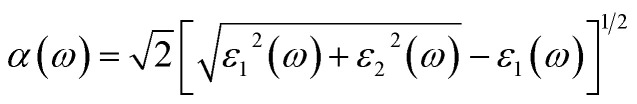
The subscripts C and V indicate the conduction band and the valence band, respectively. BZ denotes the first Brillouin zone, |*aM*_CV_(*K*)|^2^ denotes the matrix element of momentum transition, *K* denotes the wave vector of the first Brillouin zone, *ℏ* denotes Planck's constant, and *ω* denotes the angular frequency. *E*_C_(*K*) and *E*_V_(*K*) are the intrinsic energy levels on the conduction band and the valence band, respectively.

The real and imaginary part of the dielectric function *ε* = *ε*_1_ + i*ε*_2_*vs.* photon energy *hν* = 0–25 eV is plotted in Fig. 7(b). The imaginary part *ε*_2_ curve is zero below the onset of direct interband transitions between occupied and unoccupied states, and it begins to increase from *hν* > 3.23 eV. The SnO_2_ rutile is a transparent conductive oxide (TCO) that can be used as a transparent electrode for optoelectronic applications such as flat-screen displays, photovoltaic solar panels, or more generally, any device requiring electrical contact that does not reduce the passage of photons from the visible domain.

**Fig. 8:**
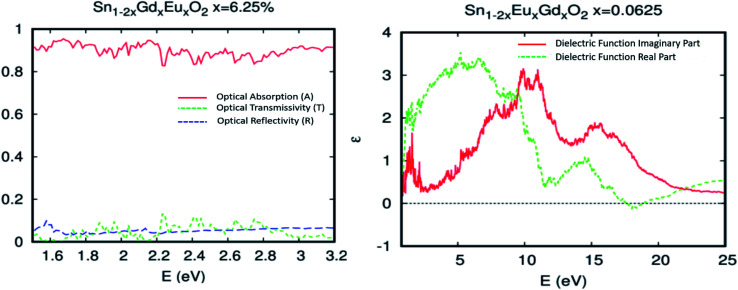
(a) The optical absorption, transmissivity, and reflectivity in visible light for Sn_1−2*x*_Gd_*x*_Eu_*x*_O_2_*x* = 0.0625, with a fixed slab thickness of *d* = 1000 nm. (b) The real and imaginary part of the dielectric function *versus* energy for Sn_1−2*x*_Gd_*x*_Eu_*x*_O_2_*x* = 0.0625.

The insertion of coupled Eu/Gd by substitution in the SnO_2_ matrix completely changes the optical properties of the parent material. The Sn_1−2*x*_Eu_*x*_Gd_*x*_O_2_ (*x* = 0.0625) becomes a ferromagnetic alloy able to absorb maximum visible light. Fig. 8(a) depicts a plot of the predicted optical absorption, transmissivity, and reflectivity *vs.* photon energy *hν* = 1.55–3.2 eV of the unit cell Sn_0.875_Gd_0.0625_Eu_0.0625_O_32_. The thickness of the slab is fixed at *d* = 1000 nm. It seems clear that the insertion of Eu/Gd in the host system has a strong influence on the optical behavior. Therefore, we have reported a high absorption under natural light.

To put it clearly, the Sn_1−2*x*_Eu_*x*_Gd_*x*_O_2_ (*x* = 0.0625) absorbed in the range of 83–96% of solar photons. In other words, we have a good correspondence between the incident solar spectrum and the band gap. The highly effective photonic conversion prompted us to conjecture what the two main mechanisms could be that are responsible for this yield. Consequently, the coupling of rare earth elements (two different ones) allowed us to convert more than visible domain photons. Also, up-conversion and down-conversion have been achieved. Within this framework of down-conversion, we have found that this DSM based on the double rare earth can also absorb in the ultraviolet (UV) light region in the range of 80–92%. In fact, the high absorption of UV photons leads to a very efficient photonic conversion process, and thus, quantum cutting is occurring.

The dielectric functions presented in Fig. 8(b), which describe the absorption of electromagnetic waves attributable to interband transitions, are presented as a curve with sharper peaks in the region of lower energy photons and the photons associated with the visible domain. It should be noted that in the case of the parent material, the curve is zero in this area. This is attributed to the difference in density of states between the undoped and codoped systems, and also to the competitive role of the 4f states associated with Eu/Gd. As a consequence, the 4f states could be an effective link of free flow charge carriers between the valence band edge and the bottom of the conduction band.

**Fig. 9:**
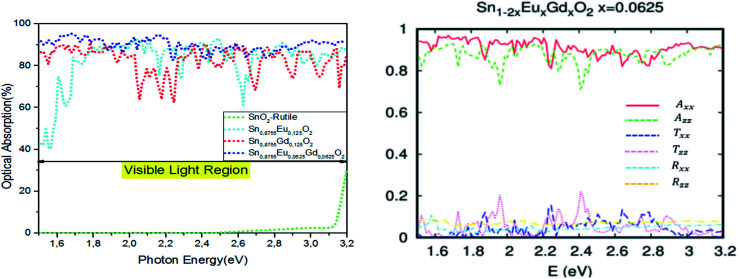
The (a) optical absorption in visible light for SnO_2_, Sn_0.875_Eu_0.125_O_2_, Sn_0.875_Gd_0.125_O_2_, and Sn_0.125_Eu_0.0625_Gd_0.0625_O_2_, with a fixed slab thickness of *d* = 1000 nm. (b) Optical absorption, transmissivity, and reflectivity in different crystallographic directions for Sn_0.9375_Eu_0.0625_Gd_0.0625_ O_2_, with a fixed slab thickness of *d* = 1000 nm.

For additional clarification on the advantage of SnO_2_ rutile doped with double rare earth elements (two different ones) rather than the traditional single rare earth element, we have collected the optical absorption curves in the visible light region for SnO_2_, Sn_0.875_Eu_0.125_O_2_, Sn_0.875_Gd_0.125_O_2_, and Sn_0.875_Gd_0.0625_Eu_0.0625_O_2_. The slab thickness is always fixed at *d* = 1000 nm. As illustrated in Fig. 9(a), the double rare earth impurities in the host matrix significantly increase the absorption in comparison with the traditional single impurity. This is attributed to the fact that quantum cutting is effective in the case of tin oxide doped with two different species of rare earth. Yet, the parent material does not absorb in the visible range.

In general, the single or double insertion of rare earth species inside a hot matrix allows quantum cutting of high-energy photons.^57–59^ Moreover, for the photon conversion process to be efficient, it requires high absorption of UV photons, which is the case for the coupled Eu–Gd that would appear to be more promising in the hot matrix of SnO_2_ rutile. Already, this couple has shown great power when inserted into a LiGdF_4_ matrix.^60^ Furthermore, Fig. 9(b) describes how the optical absorption, transmissivity, and reflectivity of Sn_14_GdEuO_32_ in visible light can be different depending on crystallographic directions. As an effect, the absorption study states that both *A*_*xx*_ and *A*_*zz*_ are almost optically isotropic. Therefore, the absorption underneath natural light, which is predominantly in the visible region, is uniform in all orientations. Thus, releasing electrons by the absorption of natural light is highly desirable to gain high power photovoltaic conversion, but it is insufficient.

For this reason, it is necessary to know how well Sn_0.875_Eu_0.0625_Gd_0.0625_O_2_ can allow the electric current to pass and conduct. Hence, the necessity of transport properties and the thermoelectric efficiency (*η*), which hinges on the thermoelectric figure of merit (*zT*) expressed as 
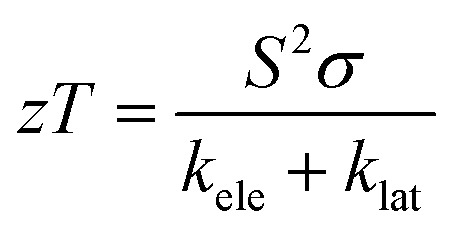
, where *S*, *σ*, *k*_ele_, *k*_lat_ and *T* denote Seebeck coefficients, electrical conductivity, electronic thermal conductivity, lattice thermal conductivity, and temperature, respectively.^61,62^

**Fig. 10:**
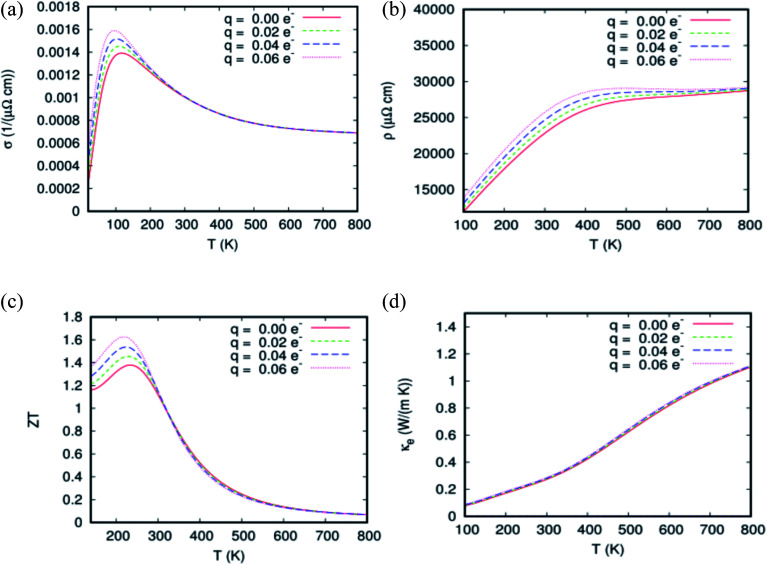
(a–d) A plot of transport properties for Sn_0.875_Eu_0.065_Gd_0.065_O_2_ as a function of doping for different temperatures. (a) Electrical conductivity, (b) electrical resistivity, (c) figure of merit (*ZT*), and (d) thermal electrical conductivity.

Fig. 10(a)–(d) exhibits a plot of transport properties as a function of doping for different temperatures. The inverse relaxation time is modeled as a linear function of temperature, and correspondingly, a zero^th^ and a first-order term can be specified: 
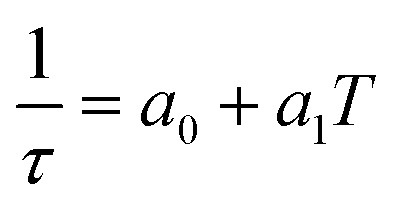
. This leads to the correct behavior of the electrical and thermal conductivity of the common metals. The electrical conductivity, *σ* of Sn_1−2*x*_Eu_*x*_Gd_*x*_O_2_, *x* = 0.0625%, is depicted in Fig. 10(a) and ensures excellent conductivity for this material. At room temperature, the *σ* value is nearly 10^5^ (Ω m)^−1^, which corresponds to a charge carrier mobility that is sufficiently high, and the dilute ferromagnetic alloy is in the range of conductors.

We also note that the value of *σ* between 50 and 150 K weakly increases with increasing doping. Also, the curves associated with different doping exhibit the same behavior in the temperature range between 200 to 800 K. However, *ρ*(*T*) of Sn_0.875_Eu_0.0625_Gd_0.0625_O_2_ given in Fig. 10(b) shows metal-like behavior with ρ300K = 2.3 × 10^−4^ Ω m. An examination of the curves reveals a low electrical resistivity for all dopings, and two regimes were observed as well: transitional for *T* ≤ 400 K; 
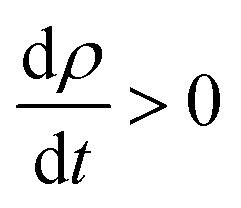
, and permanent above *T*_400K_; 
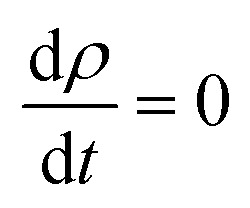
. In this context, and to confirm that the heat energy was converted into electricity, the temperature dependence of the figure of merit *zT* as a function of doping is plotted in Fig. 10(c). The value of *zT* weakly increases with increased carrier concentration in the temperature range of 100–300 K. From *T* > 300 K, the curves of the dimensionless figure of merit associated with different doping exhibit the same behavior at any given temperature. In the case of zero electrons, *zT* of the matrix reaches the peak (approximately 1.4) at 235 K and then begins to decrease to reach a value of 1.2 at 300 K.

The most optimal *zT* achieved at room temperature ensures that the material is capable of efficiently converting heat energy into electricity. Therefore, this promising thermoelectric performance is attributed to the reduction of thermal electrical conductivity by co-doping SnO_2_ rutile with double rare earth. From Fig. 10(d), the *κ*_e_ of Sn_0.875_Eu_0.0625_Gd_0.0625_O_2_ compound increases with an increase in temperature. Yet, it always remains weak, and its value at *T* = 300 K is 0.3 W m^−1^ K^−1^. Indeed, the combination of all these aspects such as the highest absorption under natural light, and the highest thermoelectric figure of merit (*zT*) in SnO_2_ rutile doped with the coupled Eu/Gd allows us to predict that the material will be powerful and highly effective at photovoltaic conversion in solar cells, and may be a key material for the future development of solar cell technology.

**Fig. 11:**
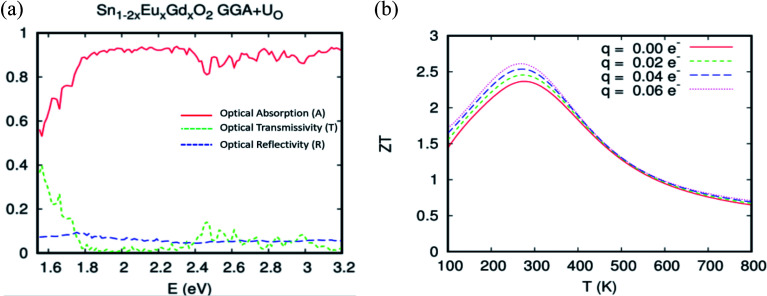
The GGA+U calculation for Sn_0.9375_Eu_0.0625_Gd_0.0625_ O_2_ with the Hubbard coefficient applied only for the anion p states of the host system for a Eu–Gd separation of 3.24 Å. (a) Optical absorption, transmissivity, and reflectivity. (b) Figure of merit (*ZT*), as a function of doping for different temperatures.

Turning now to explore the pragmatic revPBE+U approach for optical absorption in the range of 400–800 nm, we have also employed the double-counting corrections included within the fully localized limit. In the case where we applied the Hubbard coefficient only to the anion p states of the host system, the single-shot revPBE-GGA+U_O_ functional raises the gap value to 3.85 eV with an indication of a direct large half-metallic gap (Gama to Gama). As illustrated in Fig. 11(a), the optical absorption curve increases from 55% at low photon energies (*hν* = 1.55 eV) to 88% at 1.79 eV from *hν* > 1.72 eV. The curve behaves like the one achieved by the revPBE-GGA functional, which is a remarkable improvement in thermoelectric efficiency compared to that obtained by GGA, as illustrated in Fig. 11(b). The *zT* value at room temperature is 2.34 for zero carrier concentration.

**Fig. 12:**
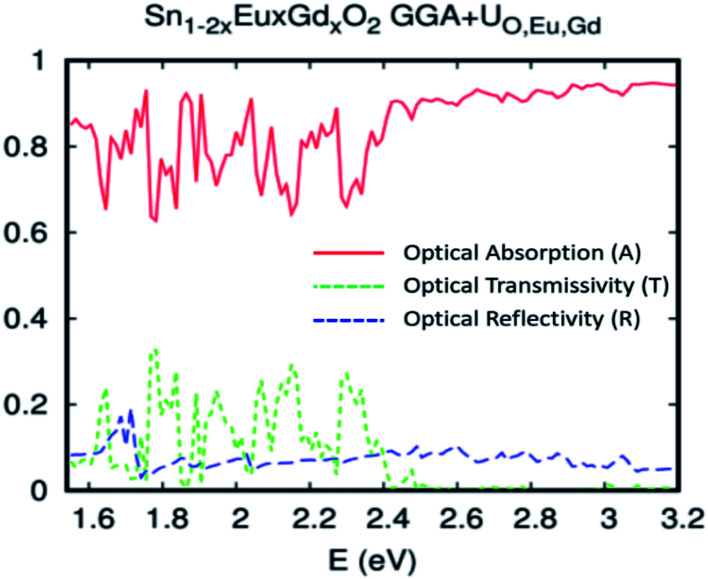
The GGA+U calculation for optical absorption, transmissivity, and reflectivity of Sn_0.9375_Eu_0.0625_Gd_0.0625_ O_2_, for a Eu–Gd separation of 3.24 Å. The Hubbard coefficient is simultaneously applied to the anion p states and 4f electrons.

The single-shot revPBE-GGA+U_O,Eu,Gd_ functional reduces the gap value at 1.65 eV with an indication of a quasi-direct Γ to X band gap: the minimum that excited at Γ and X is almost the same. The optical absorption curve indicated in Fig. 12 fluctuates between 64% and 92% in the interval photon energies *hν* = 1.55–2.4 eV. However, the strong absorption started from *hν* > 2.4 eV and reached 92%. In a nutshell, and as expected for the competitive role of the coupled Eu/Gd in the SnO_2_ host matrix, the optical absorption analysis by the different approaches used in this study roughly converge towards excellent absorption of the ferromagnetic alloy underneath natural light.

## 4 Conclusions

In this article, we systematically investigated the electronic structure, magnetic ground state, and optical properties of SnO_2_ rutile doped with double rare earth elements (two different ones) by using DFT calculations within the GGA and GGA+U method. Therefore, the rare earth couple of europium–gadolinium is most likely located at adjacent Sn sites. The half-metallic behavior of Sn_0.875_Eu_0.0625_Gd_0.0625_O_2_ is homogenous and energetically stable for different crystallographic directions.

The results also indicate that the dilute ferromagnetic alloy is capable of absorbing up to approximately 96% in the visible light region and can efficiently convert heat energy into electricity at ambient temperature. The combination of these aspects of SnO_2_ rutile doped with the couple Eu/Gd are very important for fabricating spintronic devices operating in the wavelength range of 400–800 nm that will be able to be a great source of power for high-efficiency photovoltaic conversion in solar cells.

## Conflicts of interest

There are no conflicts to declare.

## Supplementary Material

## References

[cit1] Fukumura T., Yamada Y., Toyosaki H., Hasegawa T., Koinuma H., Kawasaki M. (2004). Exploration of oxide-based diluted magnetic semiconductors toward transparent spintronic. Appl. Surf. Sci..

[cit2] Zhang S.-j., Zhang C.-w., Zhang S.-f., Ji W.-x., Li P., Wang P.-j., Li S.-s., Yan S.-s. (2017). Intrinsic Dirac half-metal and quantum anomalous Hall phase in a hexagonal metal-oxide lattice. Phys. Rev. B.

[cit3] Zhang M.-h., Zhang C.-w., Wang P.-j., Li S.-s. (2018). Prediction of high-temperature Chern insulator with half-metallic edge states in asymmetry functionalized stanene. Nanoscale.

[cit4] Ogale S. B., Choudhary R. J., Buban J. P., Lofland S. E., Shinde S. R., Kale S. N., Kulkarni V. N., Higgins J., Lanci C., Simpson J. R., Browning N. D., Sarma S. D., Drew H. D., Greene R. L., Venkatesan T. (2003). High temperature ferromagnetism with a giant magnetic moment in transparent Co-doped SnO_2−*δ*_. Phys. Rev. Lett..

[cit5] Coey J. M. D., Douvails A. P., Fitzgerald C. B., Venkatesan M. (2004). Ferromagnetism in Fe-doped SnO_2_ thin films. Appl. Phys. Lett..

[cit6] Zhang C.-w., Yan S.-s. (2009). First-principles study on ferromagnetism in Mg-doped SnO_2_. Appl. Phys. Lett..

[cit7] Fitzgerald C. B., Venkatesan M., Dorneles L. S., Gunning R., Stamenov P., Coey J. M. D., Stampe P. A., Kennedy R. J., Moreira E. C., Sias U. S. (2006). Magnetism in dilute magnetic oxide thin films based on SnO_2_. Phys. Rev. B: Condens. Matter Mater. Phys..

[cit8] Lussier A., Dvorak J., Idzerda Y. U., Ogale S. B., Shinde S. R., Choudary R. J., Venkatesan T. (2004). Comparative X-ray absorption spectroscopy study of Co-doped SnO_2_ and TiO_2_. J. Appl. Phys..

[cit9] Gopinadhan K., Pandya D. K., Kashyap S. C., Chaudhary S. (2006). Cobalt-substituted SnO_2_ thin films: A transparent ferromagnetic semiconductor. J. Appl. Phys..

[cit10] Punnoose A., Hays J., Gopal V., Shutthanandan V. (2004). Room-temperature ferromagnetism in chemically synthesized Sn_1−*x*_Co_*x*_O_2_ powders. Appl. Phys. Lett..

[cit11] Hays J., Punnoose A., Baldner R., Engelhard M. H., Peloquin J., Reddy K. M. (2005). Relationship between the structural and magnetic properties of Co-doped SnO_2_ nanoparticles. Phys. Rev. B: Condens. Matter Mater. Phys..

[cit12] Liu X. F., Sun Y., Yu R. H. (2007). Role of oxygen vacancies in tuning magnetic properties of Co-doped SnO_2_ insulating films. J. Appl. Phys..

[cit13] Fitzgerald C. B., Venkatesan M., Douvalis A. P., Huber S., Coey J. M. D., Bakas T. (2004). SnO_2_ doped with Mn, Fe or Co: Room temperature dilute magnetic semiconductors. J. Appl. Phys..

[cit14] Punnoose A., Reddy K. M., Hays J., Thurber A., Engelhard M. H. (2006). Magnetic gas sensing using a dilute magnetic semiconductor. Appl. Phys. Lett..

[cit15] Hong N. H., Sakai J., Huong N. T., Poirot N., Ruyter A. (2005). Role of defects in tuning ferromagnetism in diluted magnetic oxide thin films. Phys. Rev. B: Condens. Matter Mater. Phys..

[cit16] Misra S. K., Andronenko S. I., Reddy K. M., Hays J., Punnoose A. (2006). Magnetic resonance studies of Co^2+^ ions in nanoparticles of SnO_2_ processed at different temperatures. J. Appl. Phys..

[cit17] Hild F., Eichenberger L., Bouché A., Devaux X., Stoffel M., Rinnert H., Vergnat M. (2015). Structural and photoluminescence properties of evaporated SnO_2_ thin films doped with rare earths. Energy Procedia.

[cit18] Fakhim Lamrani A., Belaiche M., Benyoussef A., ElKenz A., Saidi E. H. (2011). First-principles study of electronic structure and magnetic properties of doped SnO_2_ (rutile) with single and double impurities. J. Magn. Magn. Mater..

[cit19] Lamrani A. F., Belaiche M., Benyoussef A., Kenz A. E. (2014). Electronic structures and ferromagnetism of SnO_2_ (rutile) doped with double-impurities: First-principles calculations. J. Appl. Phys..

[cit20] Wang Q., Pan C., Chen K., Zou S., Shen M., Su X. (2017). Efficient nanostructured quasi-single crystalline silicon solar cells by metal-catalyzed chemical etching. Sol. Energy Mater. Sol. Cells.

[cit21] Jovanov V., Moulin E., Haug F. J., Tamang A., Bali S. I. H., Ballif C., Knipp D. (2017). From randomly self-textured substrates to highly efficient thin film solar cells: Influence of geometric interface engineering on light trapping, plasmonic losses and charge extraction. Sol. Energy Mater. Sol. Cells.

[cit22] Green M. A., Emery K., Hishikawa Y., Warta W., Dunlop E. D. (2015). Solar cell efficiency table (Version 45). Prog. Photovolt: Res. Appl..

[cit23] Lin Y., Kim D. Y., Lambertz A., Ding K. (2017). Post-deposition catalytic-doping of microcrystalline silicon thin layer for application in silicon heterojunction solar cell. Thin Solid Films.

[cit24] Lokhande A. C., Chalapathy R. B. V., He M., Jo E., Gang M., Pawar S. A., Lakhande C. D., Kim J. H. (2016). Development of Cu_2_SnS_3_ (CTS) thin film solar cells by physical techniques: A status review. Sol. Energy Mater. Sol. Cells.

[cit25] Trupke T., Green M. A., Würfel P. (2002). Improving solar cell efficiencies by up-conversion of sub-band-gap light. J. Appl. Phys..

[cit26] Peper W. W., DeLuca J. A., Ham F. S. (1974). Cascade fluorescent decay of Pr^3+^ doped fluorides: achievement of a quantum yield greater emission than unity for emission of visible light. J. Lumin..

[cit27] Zhang Y., Yang W. (1998). Comment on “Generalized Gradient Approximation Made Simple”. Phys. Rev. Lett..

[cit28] Vosko S. H., Wilk L., Nusair M. (1980). Accurate spin-dependent electron liquid correlation energies for local spin density calculations: a critical analysis. Can. J. Phys..

[cit29] Williams A. R., Kübler J., Gelatt Jr C. D. (1979). Cohesive properties of metallic compounds: Augmented-spherical-wave calculations. Phys. Rev. B: Condens. Matter Mater. Phys..

[cit30] Eyert V. (2000). Basic notions and applications of the augmented spherical wave method. Int. J. Quantum Chem..

[cit31] Eyert V. , The Augmented Spherical Wave Method A Comprehensive Treatment, Lect. Notes Phys., Springer, 2007, p. 719

[cit32] Eyert V., Höck K.-H. (1998). Electronic structure of V_2_O_5_: Role of octahedral deformations. Phys. Rev. B: Condens. Matter Mater. Phys..

[cit33] Eyert V. (1996). A Comparative Study on Methods for Convergence Acceleration of Iterative Vector Sequences. J. Comput. Phys..

[cit34] Blöch P. E., Jepsen O., Andersen O. K. (1994). Improved tetrahedron method for Brillouin-zone integrations. Phys. Rev. B: Condens. Matter Mater. Phys..

[cit35] Eyert V. , The Augmented Spherical Wave Method – A Comprehensive Treatment, Lect. Notes Phys., Springer, Berlin Heidelberg, 2nd edn, 2013, p. 849

[cit36] Methfessel M. S. (1988). Elastic constants and phonon frequencies of Si calculated by a fast full-potential linear-muffin-tin-orbital method. Phys. Rev. B: Condens. Matter Mater. Phys..

[cit37] Bolzan A. A., Fong C., Kennedy B. J., Howard C. J. (1997). Structural Studies of Rutile-Type Metal Dioxides. Acta Crystallogr., Sect. B: Struct. Sci..

[cit38] Nabi Z., Kellou A., Mecabih S., Khalfi A., Benosman N. (2003). Opto-electronic properties of rutile SnO_2_ and orthorhombic SnS and SnSe compounds. Mater. Sci. Eng., B.

[cit39] Xiao W. Z., Wang L. L., Xu L., Wan Q., Zou B. S. (2009). Magnetic properties in Nitrogen-doped SnO_2_ from first-principle study. Solid State Commun..

[cit40] Dreizler M. R. and Gross K. U. E., Density Functional Theory An Approach to the Quantum Many Body Problem, Springer, Berlin, 1990, 10.1007/978-3-642-86105-5

[cit41] Themlin J. M., Sporken R., Darville J., Caudano R., Gilles J. M., Johnson R. L. (1990). Resonant-photoemission study of SnO_2_: Cationic origin of the defect band-gap states. Phys. Rev. B: Condens. Matter Mater. Phys..

[cit42] Maki-Jaskari M. A., Rantala T. T. (2001). Band structure and optical parameters of the SnO_2_(110) surface. Phys. Rev. B: Condens. Matter Mater. Phys..

[cit43] Fakhim Lamrani A. , Modélisation et Simulation par la DFT des Oxydes Magnétiques Dilués, Editions Universitaires Européennes, Détails du Livre: ISBN-13 :978-3-330-86658-4, ISBN-10: 3330866586, EAN: 9783330866584, Publié le: 11.04.2017, Nombre de pages: 156

[cit44] Baur W. H., Khan A. A. (1971). Rutile-type compounds. IV. SiO_2_, GeO_2_ and a comparison with other rutile-type structures. Acta Crystallogr., Sect. B: Struct. Crystallogr. Cryst. Chem..

[cit45] Beltran A., Andrés J., Sambrano J. R., Longo E. (2008). Density Functional Theory Study on the Structural and Electronic Properties of Low Index Rutile Surfaces for TiO_2_/SnO_2_/TiO_2_ and SnO_2_/TiO_2_/SnO_2_ Composite Systems. J. Phys. Chem. A.

[cit46] Anisimov V. I., Solovyev I. V., Korotin M. A., Czyzyk M. T., Sawatzky G. A. (1993). Density-functional theory and NiO photoemission spectra. Phys. Rev. B: Condens. Matter Mater. Phys..

[cit47] Batzill M., Diebold U. (2005). The surface and materials science of tin oxide. Prog. Surf. Sci..

[cit48] Wang X. L., Zeng Z., Zheng X. H. (2007). First-principles investigations of Co- and Fe-doped SnO_2_. J. Appl. Phys..

[cit49] Novák P., Kunes J., Chaput L., Pickett W. E. (2006). Exact exchange for correlated electrons. Phys. Status Solidi B.

[cit50] Czyzyk M. T., Sawatzky G. A. (1994). Local-density functional and on-site correlations: The electronic structure of La_2_CuO_4_ and LaCuO_3_. Phys. Rev. B: Condens. Matter Mater. Phys..

[cit51] Serin T., Serin N., Karadeniz S., Sari H., Tuğluoğlu N., Pakma O. (2006). Electrical, structural and optical properties of SnO_2_ thin films prepared by spray pyrolysis. J. Non-Cryst. Solids.

[cit52] Saikia P., Borthakur A., Saikia P. K. (2011). Structural, optical and electrical properties of tin oxide thin film deposited by APCVD method. Indian J. Phys..

[cit53] Yanlu L., Weiliu F., Honggang S., Xiufeng C., Pan L., Xian Z., Jingcheng H., Minhua J. (2010). Optical Properties of the High-Pressure Phases of SnO_2_: First-Principles
Calculation. J. Phys. Chem. A.

[cit54] Dar A., Majid A. (2015). Eur. Phys. J.: Appl. Phys..

[cit55] Luo X., Guo X., Liu Z. (2007). Phys. Rev. B: Condens. Matter Mater. Phys..

[cit56] Saha S., Sinha T. P., Mookerjee A. (2000). Phys. Rev. B: Condens. Matter Mater. Phys..

[cit57] Klampaftis E., Ross D., McIntosh K. R., Richards B. S. (2009). Enhancing the performance of solar cells *via* luminescent down-shifting of the incident spectrum: A review. Sol. Energy Mater. Sol. Cells.

[cit58] Auzel F. (2004). Upconversion and anti-Stokes processes with f and d ions in solids. Chem. Rev..

[cit59] Lamrani A. F. (2020). Rare-earth-doped TiO_2_ rutile as a promising ferromagnetic alloy for visible light absorption in solar cells: first principle insights. RSC Adv..

[cit60] Wegh R. T., Donker H., Oskam K. D., Meijerink A. (1999). Visible quantum cutting in LiGdF_4_: Eu^3+^ through downconversion. Science.

[cit61] DiSalvo F. J. (1999). Science.

[cit62] Sootsman J. R., Chung D. Y., Kanatzidis M. G. (2009). Angew. Chem., Int. Ed..

